# Phenotype variability in a large Spanish family with Alport syndrome associated with novel mutations in *COL4A3* gene

**DOI:** 10.1186/s12882-017-0735-y

**Published:** 2017-10-31

**Authors:** C. Cervera-Acedo, A. Coloma, E. Huarte-Loza, M. Sierra-Carpio, E. Domínguez-Garrido

**Affiliations:** 1 Molecular Diagnostics Unit, Center for Biomedical Research (CIBIR), Fundación Rioja Salud, C/Piqueras 98, 26006 Logroño, La Rioja Spain; 2 Department of Nephrology, San Pedro Hospital, Logroño, La Rioja Spain

**Keywords:** Alport syndrome, *COL4A3* gene, Microhematuria, Proteinuria, genotype-phenotype correlation

## Abstract

**Background:**

Alport syndrome is an inherited renal disorder characterized by glomerular basement membrane lesions with hematuria, proteinuria and frequent hearing defects and ocular abnormalities. The disease is associated with mutations in genes encoding α3, α4, or α5 chains of type IV collagen, namely *COL4A3* and *COL4A4* in chromosome 2 and *COL4A5* in chromosome X. In contrast to the well-known X-linked and autosomal recessive phenotypes, there is very little information about the autosomal dominant. In view of the wide spectrum of phenotypes, an exact diagnosis is sometimes difficult to achieve.

**Methods:**

We investigated a Spanish family with variable phenotype of autosomal dominant Alport syndrome using clinical, histological, and genetic analysis.

**Results:**

Mutational analysis of *COL4A3* and *COL4A4* genes showed a novel heterozygous mutation (c. 998G > A; p.G333E) in exon 18 of the *COL4A3* gene. Among relatives carrying the novel mutation, the clinical phenotype was variable. Two additional *COL4A3* mutations were found, a Pro-Leu substitution in exon 48 (p.P1461L) and a Ser-Cys substitution in exon 49 (p.S1492C), non-pathogenics alone.

**Conclusion:**

Carriers of p.G333E and p.P1461L or p.S1492C mutations in COL4A3 gene appear to be more severely affected than carriers of only p.G333E mutation, and the clinical findings has an earlier onset. In this way, we could speculate on a synergistic effect of compound heterozygosity that could explain the different phenotype observed in this family.

## Background

Alport syndrome (AS) is an inherited renal disorder characterized by glomerular hematuria, proteinuria, progressive renal failure, often associated with bilateral sensorineural hearing loss, and ocular abnormalities [1]. AS accounts for 0.6–2% of patients who start renal replacement therapy in Europe, with a frequency of about 1 in 5000 people, although that number is probably overestimated [2, 3]. The disease is genetically heterogeneous and associated with mutations in genes encoding α3, α4, or α5 chains of type IV collagen that forms a distinct network in the glomerular basement membrane (GBM) [4, 5]. Mutations in *COL4A5* gene located in the Xq22 and encoding the α5 (IV) chain, are responsible for the X-linked form of AS (XLAS, OMIM 301050) with an estimated frequency of 80–85% [6]. This type of AS is more severe in men than in women. Before the age of 30 years (juvenile form), 70% of affected males reach end-stage renal disease (ESRD), whereas the remaining 30% progress toward ESRD after their 30 years (rare adult form). Females with XLAS usually have only microhematuria, however, there are some cases in which they are as affected as males [7–10]. Dominant and recessive autosomal forms of AS (ADAS, OMIM 104200 and ARAS, OMIM 203780) are characterized by mutations in *COL4A3* and *COL4A4* genes, located in 2q36.3, encoding the α3 and α4 chains of IV collagen [11, 12]. The frequency estimated for ARAS and ADAS are 15% and 1–5%, respectively [13]. ARAS is associated with homozygous or compound heterozygous mutations in either *COL4A3* or *COL4A4 genes*. The disease is equally severe in males and females, and early progression toward ESRD is frequent before the end of the second decade of life [14–16]. Clinical and morphologic features are comparable to the XLAS [17]. In ADAS, both female and male patients are affected in the same way and this type shows high clinical variability, from hematuria to late onset ESRD, associated in few cases with hearing loss [15–19].

Heterozygous mutations in *COL4A4* or *COL4A3* genes have been found also in patients with benign familial hematuria (BFH, OMIM 141200), that is clinically defined by persistent glomerular hematuria and by the absence of extra-renal findings [20, 21]. The diagnosis of AS is straightforward when clinical renal and extra-renal symptoms as well as family history are typical of this disease.However, the lack of family history does not exclude the diagnosis of AS, as approximately 10% of *COL4A5* mutations occur de novo [17]. In the presence of sporadic persistent hematuria associated with proteinuria without any other symptoms suggesting AS, renal biopsy is frequently the first investigation performed. Examination of the renal tissue can exclude other hematuric glomerular disease, being the most frequent IgA nephropathy, and often shows typical ultrastructural abnormalities of the GBM when electron microscopy (EM) is performed [17].

The identification of the mode of inheritance of AS is essential for providing genetic counselling to affected families. Clinical and family history together with morphologic phenotypes do not always allow to conclude the type of transmission of the disease. The analysis of the type IV collagen chains expression in skin and/or kidney basement membranes can make it easier [22]. However, the expression of these collagen chains is normal in about one third of the families, and it does not exclude the diagnosis at all. In these cases, molecular testing might be required to make a definitive diagnosis [23]. The identification of new mutations and their correlation with clinical phenotype are important to offer an early treatment and familiar genetic counselling.

Here, we report the analysis of the *COL4A3* and *COL4A4* genes in a Spanish family with variable clinical phenotype of ADAS.

## Methods

**Fig. 1:**
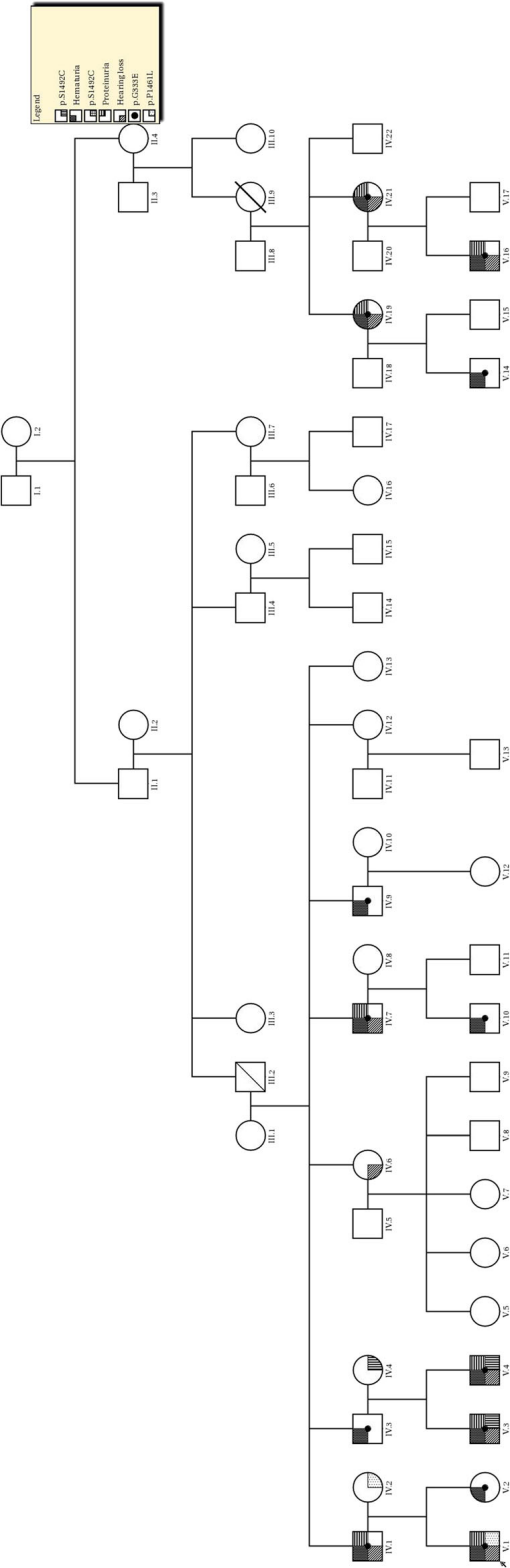
Pedigree of the family where mutations of *COL4A3* gene were found. Squares are males and circles are females. An oblique bar indicates a deceased individual. The arrow indicates the proband patient

This study was conducted according to protocols approved by the Ethical Committee of La Rioja (CEICLAR PI-161), and written informed consent was obtained from each patient for publication. The proband and 18 members of the family (11 males and 8 females), with clinical suspect of AS, were included in this study. Familiar clinical history was performed carefully in each case by reconstruction of the pedigree for at least three generations (Fig. 1). Urinalysis and renal function evaluations were carried out in all patients. Audiological and ophthalmic examinations were performed, and, whenever indicated, a renal biopsy was analysed by LM and the ultrastructural study was performed by EM.

Proband (V.I) is a 31-year-old male patient that was referred to the Pediatric Nephrology Service at the age of 8 years for evaluation of persistent hematuria.

He fulfilled three of the AS clinical diagnostic criteria: a positive family history of hematuria, proteinuria and hearing loss. Ophthalmic evaluation was normal. The results of audiometric testing revealed a bilateral sensorineural hearing loss. At the age of 13 years, urine analysis revealed proteinuria (1.77 g/24 h), so he underwent a renal biopsy. LM revealed minimal mesangial changes, with no glomeruli or tubulointerstitial lesions, and the immunofluorescence microscopy was negative for IgA, IgG, IgM, C3 and C4. He started treatment with inhibitors of angiotensin converting enzyme (ACE inhibitors). Nevertheless, progressive increase in proteinuria levels (up to 5.2 g/24 h) with clinical and biochemical nephrotic syndrome were found. For this reason, a second renal biopsy was performed. The results were compatible with thin basement membrane disease (TBMD). Antiproteinuric treatment was reinforced with ACE inhibitors and spironolactone. Due to the persistence of nephrotic syndrome, tacrolimus treatment was started, but no response after 10 months of treatment was obtained. At the age of 25 years, he started with progressive renal failure and three years later he received renal transplantation from living related donor. Considering the clinical course of the patient, and keeping in mind the hearing impairment and the family history, molecular genetic testing was carried out. This study was extended to all of family members (10 males and 8 females, age ranges from 25 to 66 years). In order to determine the molecular cause of the disease, blood samples from the proband and his family were collected after genetic counseling and informed consent. Genomic DNA and RNA were isolated from EDTA peripheral blood samples using QIAamp DNA Blood kit and RNeasy Mini kit, respectively, according to the manufacturer’s protocol (Qiagen, http://www.qiagen.com). Mutation analysis of *COL4A3* (NM_000091) and *COL4A4 (NM_000092)* genes was performed by analyzing the entire coding sequences and flanking intronic regions by PCR and subsequent Sanger sequencing in both directions. PCR products were purified with ExoStar™ (GE Healthcare). Sequencing reactions were performed using Big Dye® Terminator v.3.1 Cycle Sequencing kit (Applied Biosystems, Forest City, CA, USA), and purified with Big Dye X-Terminator (Applied Biosystems, Foster City, CA, USA). DNA sequences were analyzed on an ABI PRISM® 3130 automated sequencer (PE Applied Biosystems, Forest City, CA, USA) with Sequencing Analysis Software (Applied Biosystems, Foster City, CA). After that, the obtained sequences were analyzed using SeqScape v2.5 software. The specific primer sequences are available upon request. Pathogenicity of the detected variants was predicted by in silico analysis with bioinformatics tools such as Sorting Intolerant From Tolerant (SIFT), Mutation Taster, Polyphen-2, and Human Splicing Finder (HSF 3.0). ExAC browser of Broad Institute, 1000 Genomes database and dbSNP138.

## Results

### Renal morphological findings

**Fig. 2:**
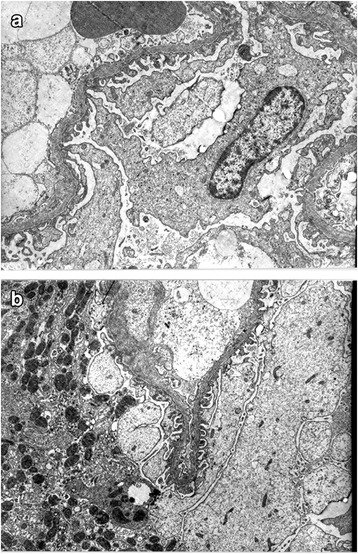
Electron microscopy of proband kidney biopsy. **a** Glomeruli ultrastructure with thickening of the GBM. Note the division and lamelation of BM (×6500). **b** Focal fusion of podocyte foot, and reduction of GBM thickness between 150 and 217 nm (×8000)

In the first biopsy from the proband (V.1), LM revealed minimal abnormalities in the mesangial area with normal glomeruli, tubules and interstitium. In the second one, LM showed lesions with areas of tubular atrophy, focal interstitial fibrosis and a slight lymphoid infiltrate, without changes in the GBM or vascular damage. Masson’s trichrome staining revealed glomeruli with segmental hyalinization. Thinning, characteristically pronounced thickening of the GBM and weakened podocyte adhesion to the GBM were found by EM (Fig. 2). These findings were compatible with the diagnosis of TBMD.

### Genetic analysis

Analysis of all exons of the *COL4A3* and *COL4A4* genes revealed the presence of a novel heterozygous mutation c. 998G > A in exon 18 resulting in p.G333E of the *COL4A3* gene. No mutations were found in *COL4A4* gene. p.G333E mutation was found in the proband and it was confirmed in 12 members of the family (9 males and 3 females). The mutation was absent in 100 healthy controls. We used PolyPhen and SIFT to predict possible functional effects, and the variant was classified as possibly damaging. No further pathogenic *COL4A3* mutations were detected in this family. A Pro-Leu heterozygous substitution in exon 48 (c.4382C > T; p.P1461L) was found in the proband (V.1), his mother (IV.2) and his sister (V.2), and a Ser-Cys heterozygous substitution in exon 49 (c.4474A > T; p.S1492C) was found in two proband’s cousins (V.3 and V.4) and in his aunt in law (IV.3), both in *COL4A3* gene, with an unclear pathogenicity. These mutations have not been described before and were absent in 100 healthy controls.

### Genotype-phenotype correlation

Detailed clinical information of the 19 patients belonging to this family has been collected in Table 1. Regarding the clinical manifestations associated with the p.G333E mutation, hematuria was found in 100% of cases, while hearing loss and proteinuria were presented in 6/13 (46.1%) and 8/13 (61.5%) patients, respectively. Nephrotic-range proteinuria was observed in 4/13 (30.7%) of the patients, 3 males and 1 female. 75% of patients with proteinuria in the nephrotic range showed renal failure, and it accounts for 23% of the carriers. Renal biopsy was obtained from 1 of these males (the proband), and the results were compatible with the diagnosis of focal segmental glomerulonephritis (FSGS). Ocular lesions were not identified in any case. With respect of the patients with compound heterozygous *COL4A3* mutations, p.G333E and p.P1461L (V.1) or p.G333E and p.S1492C (V.3 and V.4), the phenotype was more severe, with an early upset of the disease, and reaching nephrotic syndrome. In addition, V.1 and V.3 patients received a renal transplant from their mothers. On the other hand, the patients carrying only p.P1461L or p.S1492C mutations (IV.2 and IV.4), did not present any clinical manifestations.

**Table 1:** Clinical and molecular data of reported patients

Patient	Gender	Age	Hematuria	Proteinuria	Renal Function	Hearing loss	Ocular lessions	*COL4A3* Mutations
IV.1	M	58	Yes	1.08 g/24 h	Normal	Yes	No	c.998G > A; p.G333E
IV.2	F	56	No	No	Normal	No	No	c.C4382T; p.P1461L
IV.3	M	62	Yes	No	Normal	No data	No data	c.998G > A; p.G333E
IV.4	F	66	No	No	Normal	No	No data	c.A4474T; p.S1492C
IV.6	F	57	No	No	Normal	Yes	No data	N.D.
IV.7	M	54	Yes	1.26 g/24 h	Normal	Yes	No	c.998G > A; p.G333E
IV.9	M	47	Yes	No	Normal	No data	No data	c.998G > A; p.G333E
IV.12	F	50	No	No	Normal	No	No	N.D.
IV.19	F	56	Yes	>3.5 g/24 h	CRF-dialisis	Yes	No	c.998G > A; p.G333E
IV.21	F	53	Yes	0.9 g/24 h	Normal	Yes	No	c.998G > A; p.G333E
V.1	M	31	Yes	>3.5 g/24 h	CRF-renal transplant	Yes	No	c.998G > A; p.G333E + c.C4382T; p.P1461L
V.2	F	27	Yes	No	Normal	No	No	c.998G > A; p.G333E
V.3	M	34	Yes	>3.5 g/24 h	CRF-renal transplant	Yes	No	c.998G > A; p.G333E + c.A4474T; p.S1492C
V.4	M	32	Yes	>3.5 g/24 h	Nephrotic Syndrome	Yes	No	c.998G > A; p.G333E + c.A4474T; p.S1492C
V.5	F	25	No	No	Normal	No data	No data	N.D.
V.10	M	27	Yes	No	Normal	No	No	c.998G > A; p.G333E
V.11	M	34	No	No	Normal	No	No	N.D.
V.14	M	25	Yes	No	Normal	No data	No	c.998G > A; p.G333E
V.16	M	26	Yes	0.35 g/24 h	Normal	Yes	No data	c.998G > A; p.G333E

*CRF* Cronic renal failure, *F* female, *M* male, *N.D.* not detected

## Discussion

ADAS is a genetically and phenotypically heterogeneous disease. XLAS is, by far, the commonest, although ADAS and ARAS have also been described and are said to be the mode of inheritance in 15% of families [24]. Mutations in *COL4A3, COL4A4*, or *COL4A5* genes result in a spectrum of phenotypes from BFH to AS [11], including XLAS, ARAS, or ADAS. Accurate genetic counseling and reasonable estimation of prognosis in patients and families with persistent hematuria require precise diagnosis. Comprehensive clinical evaluation and pedigree analysis (including testing of relatives for hematuria) combined with histopathology and study of type IV collagen expression in skin and kidney base membrane are sometimes sufficient to firmly establish a diagnosis and determine the risk of transmission. However, in the majority of cases, molecular analysis is necessary to achieve the desired level of diagnostic accuracy. Mutations in *COL4A3* and *COL4A4* genes have been found to be involved in the pathogenesis of ARAS and ADAS, as well as of TBMD, with a high clinical variability [11, 25]. Most *COL4A3* and *COL4A4* mutations have been detected to cause ARAS, and only a small number of mutations in those genes have been described for TBMD or ADAS [26]. They are also associated with a wide spectrum of phenotypes ranging from isolated microhaematuria to ESRD, as highlighted also by the intra- and inter-clinical variability reported in ADAS. In this form, male to male transmission can be observed, the renal disease progresses in slow way in males as well as in females and, when documented, the EM shows thick, thick and thin, or diffuse thin GBM. The hearing loss, when present, is a late event [17]. The clinical features of ADAS are similar to those of X-linked disease. However, deterioration of renal function tends to occur more slowly [12, 27, 28].

In this family, pedigree analysis suggested a dominant inheritance pattern. All exons and their intronic boundaries of *COL4A3* and *COL4A4* genes were analyzed and a novel pathogenic heterozygous mutation in *COL4A3* gene (c. 998G > A; p.G333E) was found. All affected patients carried this mutation. The course of the nephropathy was more severe, with earlier onset in the proband and his cousin compared to their fathers. We found that they carried an aditional *COL4A3* mutation on the allele inherited from his mother. The Pro-Leu substitution in exon 48 and the Ser-Cys substitution in exon 49 on one of the maternal chromosomes are not pathogenic in itself as it is reflected in the phenotype of the patients, but may have a secondary effect on the mutated *COL4A3* chain. As the carriers of p.G333E and p.P1461L or p.S1492C mutations appear to be more severely affected than the carrier of only p.G333E mutation, we could speculate on a synergistic effect of compound heterozygosity. However, the possibility of such an effect has been reported in the literature previously [16]. For example, in the case of dystrophic epidermolysis bullosa, a neutral glycine substitution in the *COL7A1* gene increased the effect of a dominant glycine substitution in the other copy of the *COL7A1* gene, resulting to a more severe phenotype in the compound heterozygote [18].

This family demonstrates that one mutation alone cannot explain the highly variable renal phenotype. We might suppose that the effect of a pathogenic mutation could be influenced by the presence of certain variants in the same genes and/or in other genes that are key players in renal filtration [12]. This is the case of a family with XLAS, in which the coinheritance of *COL4A5* mutations and homozygous *MYO1E* variants were associated with more severe kidney disease [29], and can explain a highly variable renal phenotype, inexplicable by conventional pedigree analysis. As described in this paper, the screening for *MYO1E* or other non-*COL4* podocyte gene mutations in XLAS is suggested when clinical nephropathy is more severe than expected. The dominant nature of *COL4A3* mutations and the different clinical manifestations can be explained in several ways. The integration of the altered collagen chain in the final network, thereby disturbing normal structure and functioning [23]. The synthesis of a theoretically equal number of normal an abnormal chains would result in production of a 1:1 ratio of abnormal to normal molecules. Alternatively, the shortened *COL4A3* chain may not be incorporated at all. The shortened protein is not integrated in trimers, nor is secreted [20] but still has a dominant effect, probably because of a reduction in the level of the protein. Therefore, the nature of the defect, with respect to position and function of the altered or deleted amino acids, and the specific chain involved, either *COL4A3* or *COL4A4*, may determine the phenotypic outcome as well.

The main clinical manifestation of ADAS is hematuria followed by proteinuria and progressive renal failure [12, 29]. Proteinuria is variable and can be in the nephrotic range, but rarely resulting in nephrotic syndrome. In our family, we observed microscopic hematuria in 100% of p.G333E carrier patients, and 75% of patients with proteinuria in the nephrotic range developed clinical and biochemical nephrotic syndrome.

In contrast with the other forms of AS, loss of renal function is gradual [12, 30]. In our study, the renal failure progression was variable; 70% of p.G333E carriers developed ESRD before the age of 30, while the remaining patients developed it later. We also observed that proteinuria levels greater than 3,5 g/24 h was associated with worst evolution, and unresponsive to treatment with ACE inhibitors, angiotensin II receptor blockers (ARB) [31, 32] or calcineurin inhibitors, such as Tacrolimus [33].

Regarding histological findings, biopsy from the proband revealed areas of thinning of the GBM so a diagnosis of TBMD was done. Thinning of the GBM is non-specific and can be appeared both in BFH and AS. TBMD is recognized as the leading cause of microhematuria and is regarded as a benign condition [34]. Characteristic AS hearing loss occurs in 70% of males before 40 years and in 45% of females with XLAS [8]. Hearing loss is never congenital and it has been always described associated with renal disorder. Its real incidence could be underestimated by lack of audiological examination. For ADAS, hearing loss is not very common, and is around 13 and 27%, based on the studies published [12, 30]. In our family, we observed hearing loss in 46.1% of patients carrying p.G333E mutation.

Typical AS ocular lesions are not present in patients with ADAS in contrast to XLAS and ARAS [30]. These results were also confirmed in our family.

The increasing use of global and unbiased genetic analysis with next-generation sequencing is likely to reveal disease-modifying variants across the range of human genetic disease [29]. The detection of a mutation in affected family members is also important for prenatal diagnosis and subsequent diagnostics of their offspring and could therefore reduce the need for more invasive renal biopsies [35]. The role of molecular diagnosis combined with the mode of transmission may be important for the assessment of the prognosis of the disease, for the differentiation of patients with either TBMD or AS, and may represent a future diagnostic approach [36].

## Conclusion

It is very difficult to predict the prognosis in a patient with a heterozygous mutation in either the *COL4A3* or the *COL4A4* genes. In our study, carriers of p.G333E and p.P1461L or p.S1492C mutations in *COL4A3* gene appear to be more severely affected than carriers of only p.G333E mutation, and the clinical findings has an earlier onset. In this way, we could speculate on a synergistic effect of compound heterozygosity that could explain the different phenotype observed in this family. A correct diagnosis and prognosis should be based on a combination of a comprehensive clinical investigation of all family members, including examination of renal and extra-renal signs of AS, associated with a broadly formal molecular genetic analysis of the pedigree.

## Abbreviations

ACE: Inhibitors, angiotensin converting enzyme
ADAS: Dominant autosomal form of AS
ARAS: Recessive autosomal form of AS
ARB: Angiotensin II receptor blockers
AS: Alport syndrome
BFH: Benign familial hematuria
EM: Electron microscopy
ESRD: End-stage renal disease
FSGS: Focal segmental glomerulonephritis
GBM: Glomerular basement membrane
LM: Light microscopy
SIFT: Sorting Intolerant From Tolerant
TBMD: Thin basement membrane disease
XLAS: X-linked form of AS
